# Draft Genome Sequence of *Clostridium* sp. Strain Ade.TY, a New Biohydrogen- and Biochemical-Producing Bacterium Isolated from Landfill Leachate Sludge

**DOI:** 10.1128/genomeA.00078-14

**Published:** 2014-03-06

**Authors:** Y. M. Wong, J. C. Juan, Adeline Ting, T. Y. Wu, H. M. Gan, C. M. Austin

**Affiliations:** a School of Science, Monash University Malaysia, Jalan Lagoon Selatan, Bandar Sunway, Malaysia; b Nanotechnology & Catalysis Research Centre (NANOCAT), University of Malaya, Kuala Lumpur, Malaysia; c Chemical Engineering Discipline, School of Engineering, Monash University, Jalan Lagoon Selatan, Bandar Sunway, Malaysia

## Abstract

*Clostridium* sp. strain Ade.TY is potentially a new biohydrogen-producing species isolated from landfill leachate sludge. Here we present the assembly and annotation of its genome, which may provide further insights into its gene interactions for efficient biohydrogen production.

## GENOME ANNOUNCEMENT

The use of fossil fuel as the primary energy source causes various adverse side effects, including air pollution and climate change. Therefore, there is a need to find sources of clean, renewable energy (1, 2). Hydrogen is an environmentally safe energy source because the combustion of hydrogen produces water as the only end product. Most importantly, hydrogen can be produced biologically from organic waste and hence it is an ideal source of clean, renewable energy.

Hydrogen-producing bacteria that live in extreme environments such as hazardous and nutrient-scarce conditions have industrial significance because they are less susceptible to external stress (3, 4). In this work, we aimed to isolate novel hydrogen-producing bacteria from landfill leachate sludge. Since landfills and their sludge are constantly nutrient limited, hydrogen-producing bacteria that survive in these environments can adapt to harsh conditions and they possess unique features for biohydrogen and biochemical production.

Members of the genus *Clostridium* are obligate anaerobes that produce hydrogen more efficiently than do facultative anaerobes, such as *Bacillus* sp., *Klebsiella* sp., and *Enterobacter* sp. (5–11). In addition, all production of biological hydrogen is accompanied by the production of useful organic acids and solvents, such as acetate, butyrate, lactate, formate, ethanol, and butanol, which have industrial applications. Hence, *Clostridium* spp. have promising potential applications in industrial biotechnology.

The genome sequencing of *Clostridium* sp. strain Ade.TY was performed using the Illumina MiSeq Benchtop Sequencer (2 × 150-bp paired-end sequencing). The reads were trimmed and assembled *de novo* using CLC Genomics Workbench 6.0 (CLC Bio, Denmark). Multiple genome alignment was conducted using Gegenees 2.0.3. The average similarities of the conserved core and the size of the core were set at 20% (12). The genome sequence was annotated with the Rapid Annotations using Subsystems Technology (RAST) 4.0 server (13). RNAmmer 1.2 and tRNA-scan-SE 1.21 were used to predict rRNA and tRNA, respectively (14, 15). Based on 16S rRNA analysis, strain Ade.TY has a 99% identity score with several uncultured bacteria strains, and the 16S-rRNA phylogenetic tree also revealed that *Clostridium* sp. strain Ade.TY is a branch that is distant from other *Clostridium* species. This finding suggests that *Clostridium* sp. strain Ade.TY may be a new hydrogen-producing species. This is further demonstrated by the heat plot from multiple-genome alignment, which revealed that strain Ade.TY has <50% similarity to the existing complete and draft genome databases of *Clostridium* species. The draft genome sequence comprises 3,113,901 bases in 66 contigs. It has a GC content of 26.75% and contains 3,104 genes and 9 rRNAs and 68 tRNAs.

*Clostridium* sp. strain Ade.TY contains a dimeric-periplasmic [Fe] hydrogenase and two [Ni–Fe] hydrogenases. It has an energy-converting hydrogenase that is regulated by six gene clusters, *HypA*, *HypB*, *HypC*, *HypD*, *HypE*, and *HypF*, and a dimeric [Ni-Fe] hydrogenase (16).

### Nucleotide sequence accession numbers.

This whole-genome shotgun project has been deposited at DDBJ/EMBL/GenBank under the accession number AVSV00000000. The version described in this paper is version AVSV01000000.
